# Folic acid supplementation alleviates reduced ureteric branching, nephrogenesis, and global DNA methylation induced by maternal nutrient restriction in rat embryonic kidney

**DOI:** 10.1371/journal.pone.0230289

**Published:** 2020-04-06

**Authors:** Midori Awazu, Mariko Hida

**Affiliations:** Department of Pediatrics, Keio University School of Medicine, Shinjuku-ku, Tokyo, Japan; Max Delbruck Centrum fur Molekulare Medizin Berlin Buch, GERMANY

## Abstract

We previously reported that maternal nutrient restriction (NR) inhibited ureteric branching, metanephric growth, and nephrogenesis in the rat. Here we examined whether folic acid, a methyl-group donor, rescues the inhibition of kidney development induced by NR and whether DNA methylation is involved in it. The offspring of dams given food *ad libitum* (CON) and those subjected to 50% food restriction (NR) were examined. NR significantly reduced ureteric tip number at embryonic day 14, which was attenuated by folic acid supplementation to nutrient restricted dams. At embryonic day 18, glomerular number, kidney weight, and global DNA methylation were reduced by NR, and maternal folic acid supplementation again alleviated them. Among DNA methyltransferases (DNMTs), DNMT1 was strongly expressed at embryonic day 15 in CON but was reduced in NR. In organ culture, an inhibitor of DNA methylation 5-aza-2 '-deoxycytidine as well as medium lacking methyl donors folic acid, choline, and methionine, significantly decreased ureteric tip number and kidney size mimicking the effect of NR. In conclusion, global DNA methylation is necessary for normal kidney development. Folic acid supplementation to nutrient restricted dams alleviated the impaired kidney development and DNA methylation in the offspring.

## Introduction

It is now well recognized that an adverse intrauterine environment affects the health and disease in the adulthood referred to as the developmental origins of health and disease (DOHaD) concept. With respect to the kidney, low birth weight is associated with a risk for hypertension and renal disease. Reduced nephron number has been linked to these associations. In animals, maternal protein or global nutrient restriction, uterine artery ligation, and exposure to various agents such as glucocorticoids or alcohol produce offspring with fewer nephrons [1–5]. As a mechanism of reduced nephron endowment, we previously reported reduced ureteric bud branching in a rat model of maternal nutrient restriction [6].

One of the proposed mechanisms of DOHaD is epigenetic modification. DNA methylation is a most extensively studied epigenetic modification generally suppressing gene expression. Maternal nutrient restriction has been shown to change global DNA methylation of various organs including the kidney [7]. DNA methylation is regulated by DNA methyltransferases (DNMTs). DNMT1 is essential for the maintenance of DNA methylation, and DNMT 3A and 3B are required for *de novo* methylation. DNMTs catalyze the transfer of a methyl residue from the methyl donor S-adenosyl-methionine (SAM) to the 5 position of the cytosine ring. For the generation of SAM, nutrients including folic acid (FA), choline, methionine, and vitamin B12 are necessary and referred to as methyl donors.

Maternal FA supplementation has been shown to prevent hypertension induced by maternal protein restriction [8]. The mechanism of lowered blood pressure remains unknown. Of note, maternal low protein diet causes reduced nephron number that is known to be associated with hypertension [1, 9]. It is conceivable that the mechanism of the prevented hypertension was by an alleviation of reduced nephron number by maternal FA supplementation.

In the present study, we examined whether FA supplementation rescues kidney development inhibited by maternal nutrient restriction. We also examined the role of DNA methylation in normal kidney development.

## Materials and methods

### Experimental animals

Approval was obtained from the Animal Experiment Committee of Keio University. Female Sparague Dawley rats at day 1 of pregnancy were purchased from Sankyo Labo Service corporation, Inc. (Tokyo, Japan). The offspring of dams given food *ad libitum* (CON, n = 16 litters) and those subjected to nutrient restriction throughout pregnancy (NR, n = 16 litters) were examined. Nutrient-restricted dams were given half amount of chow consumed by control rats on the previous day. A separate group of dams were given half amount of chow supplemented with FA 5 mg/kg/day during pregnancy (NRFA, n = 10 litters). Dams were anesthetized with intraperitoneal injection of medetomidine hydrochloride, midazolam, and butorphanol tartrate, and fetuses were delivered by Cesarean section. Dams were then euthanized with pentobarbital. Kidneys from fetuses were fixed in cold methanol for whole mount staining, frozen for future DNA methylation analysis, lysed for immunoblotting, or fixed in 4% paraformaldehyde for hematoxylin and eosin staining. For organ culture, 13-day pregnant rats fed freely were used. Embryos of both sexes were used without distinction in all experiments since previous studies reported that maternal undernutrition reduced nephron number in both sexes [10].

### Reagents

FA (F8758) and 5-aza-2'-deoxycytidine (Aza) were from Sigma (St. Louis, MO). The following primary antibodies were used: DNMT1 (D63A6, #5032) and DNMT3A (#2160) from Cell Signaling Technology (Danvers, MA), Dnmt3b (EPR3523) from GeneTex (Irvine, CA), pancytokeratin (C2562) and α tubulin (T9026) from Sigma (St. Louis, MO), and Six2 polyclonal antibody (11562-1-AP) from proteintech (Rosemont, IL). Horseradish peroxidase-conjugated anti-mouse IgG (NA931) and anti-rabbit IgG (NA934) were from Amersham (Buckinghamshire, UK). Cy3-conjugated rabbit anti-mouse antibody was from Chemicon (AP160C, Temecula, CA). Fluorescein goat anti-rabbit antibody (Fl-1000) was from Vector Lab (Burlingame, CA). SlowFade Antifade Kit was from Molecular Probes, Inc. (Eugene, OR). Dulbecco’s Modified Eagle Medium (DMEM), fetal bovine serum (FBS), penicillin, streptomycin, and Hanks’ Balanced Salt Solution were from GIBCO Laboratories (Grand Island, NY). DMEM without FA was from Sigma (D2429, St. Louis, MO). DMEM lacking FA, choline, and methionine was custom ordered from Nacalai Tesque, Inc (Kyoto, Japan).

### Whole mount staining

Metanephroi were fixed with cold methanol for 10 min, washed three times with phosphate-buffered saline/0.1% Tween 20 (PBT), and incubated with anti-pancytokeratin antibody (dilution 1:400) for 2 h followed by incubation with Cy3-conjugated anti-mouse antibody (dilution 1:400) for 1 h. Samples were washed three times with PBT, mounted in SlowFade, and viewed under a confocal imaging system (Leica TCS-SP5, Leica Microsystems, Tokyo, Japan). Kidney size was assessed by measuring planar surface area using ImageJ software (National Institutes of Health, Bethesda, Maryland). Embryonic kidney surface has been shown to correlate with volume [11].

### Glomerular number

Glomerular number was counted as previously described [12]. Sections of 8 μm were cut from paraffin-embedded kidneys. Because the glomerular diameter equals approximately 60 μm, every fourth section was stained with PAS. Glomeruli from the S-shaped body stage onward on all sections were counted.

### DNA methylation

DNA was extracted from E15 and E18 metanephroi using DNA Zol (Invitrogen, La Jolla, CA). DNA methylation was quantified using Imprint® methylated DNA quantification kit (Sigma, St. Louis, MO) according to the manufacturer's instructions.

### Immunoblot analysis

Kidneys were lysed in solubilization buffer containing 20 mM HEPES (pH 7.2), 1% Triton X-100, 10% glycerol, 20 mM sodium fluoride, 1 mM sodium orthovanadate, 1 mM PMSF, 10 μg/mL aprotinin, and 10 μg/mL leupeptin. Insoluble material was removed by centrifugation (10,500 *g*, 10 min). Lysates (60 μg) were resolved by SDS-PAGE and transferred to PVDF membranes (Immobilon, Millipore, Bedford, MA). Nonspecific binding sites were blocked in TBS buffer (10 mM Tris・HCl, pH 7.4, 0.15 M NaCl) containing 0.1% Tween 20 and 5% BSA overnight at 4ºC or for 1 h at room temperature. Antibodies were added to TBS containing 0.1% Tween 20 with 5% BSA and incubated with mixing for 24 h at 4°C or for 2 h at room temperature. After incubating with secondary antibody, bound antibodies were detected using the ECL Western blotting system (Amersham, Arlington Heights, IL). All experiments were repeated at least three times on separate samples.

### Metanephric organ culture

Culture of embryonic kidneys was performed using low-volume system [13]. Embryonic day 13 (E13) metanephroi were isolated from normally fed dams and cultured in sterilized silicone rings on coverslips with 85 μl DMEM containing 10% FBS with vehicle or an inhibitor of DNA methylation Aza 20 μM. In separate experiments, metanephroi were cultured in DMEM, DMEM deficient in FA, or DMEM deficient in FA, choline, and methionine, containing 10% FBS for 3 days. Medium was changed every day. After 3 days, metanephroi were fixed in cold methanol for whole mount staining with anti-pancytokeratin antibody.

### Statistical analysis

The results were expressed as means±SE. Statistical analysis was performed with unpaired *t* test or ANOVA followed by Tukey’s test. Statistical significance was determined as P<0.05.

## Results

### Effect of folic acid supplementation on the reduction in body weight and ureteric tip number induced by maternal nutrient restriction at embryonic day 14

**Fig 1:**
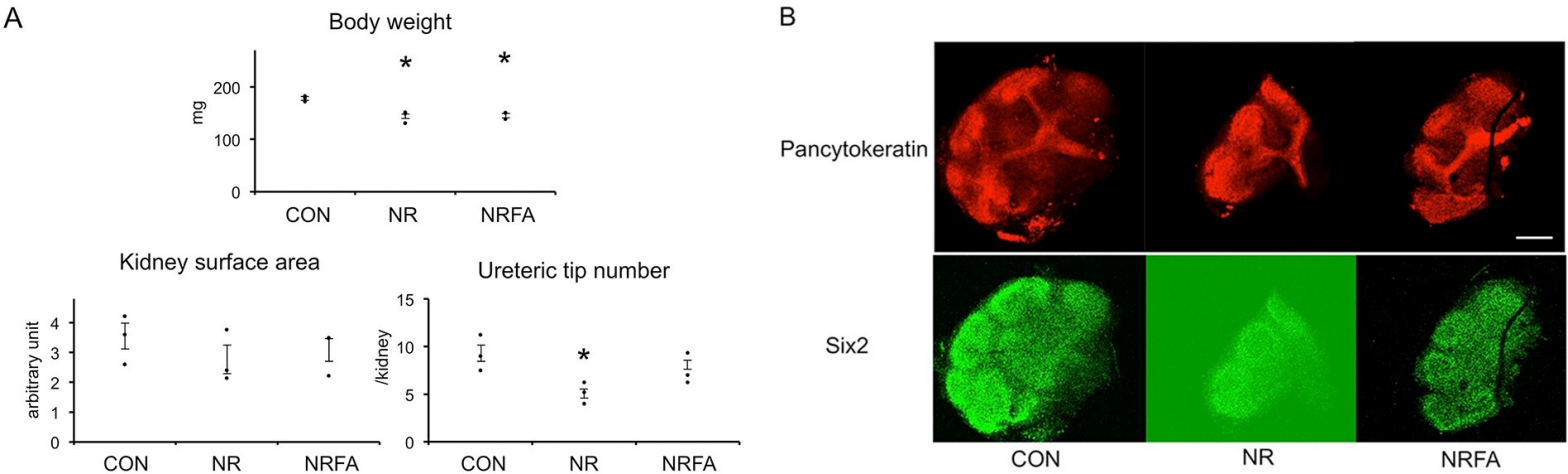
Effect of folic acid supplementation on the reduction in body weight and ureteric tip number induced by maternal nutrient restriction at embryonic day 14. (A) After fetal weight was recorded, kidneys were isolated, fixed with methanol, and stained with anti-pancytokeratin antibody and anti-Six2 antibody. The number of ureteric bud tips was counted observing z-stack images by confocal microscopy. Kidney surface area was measured by ImageJ software and expressed in arbitrary units. The mean value of each litter was used for statistical analysis (ANOVA followed by Tukey’s test). CON, controls; NR, offspring of nutrient-restricted mothers; NRFA, offspring of nutrient-restricted mothers supplemented with folic acid. n = 3 litters, n = 4–6 animals per litter. Dots indicate the mean value of each litter. Bars represent ±SE. *, P<0.05 vs CON. (B) Representative images. Scale bar, 250 μm.

Maternal nutrient restriction significantly reduced fetal body weight and ureteric tip number at E14 in agreement with our previous report (Fig 1)[6]. Kidney surface area was not changed. FA supplementation did not affect body weight, but alleviated the reduction in ureteric tip number (*P*<0.05). A marker of nephron progenitors, Six2 was observed around the ureteric tips, and decreased by NR. In NRFA, in correlation with ureteric tip number, Six2 expression was partially restored.

### Effect of folic acid supplementation on the reduction in body weight, kidney weight, and glomerular number induced by maternal nutrient restriction at embryonic day 18

**Fig 2:**
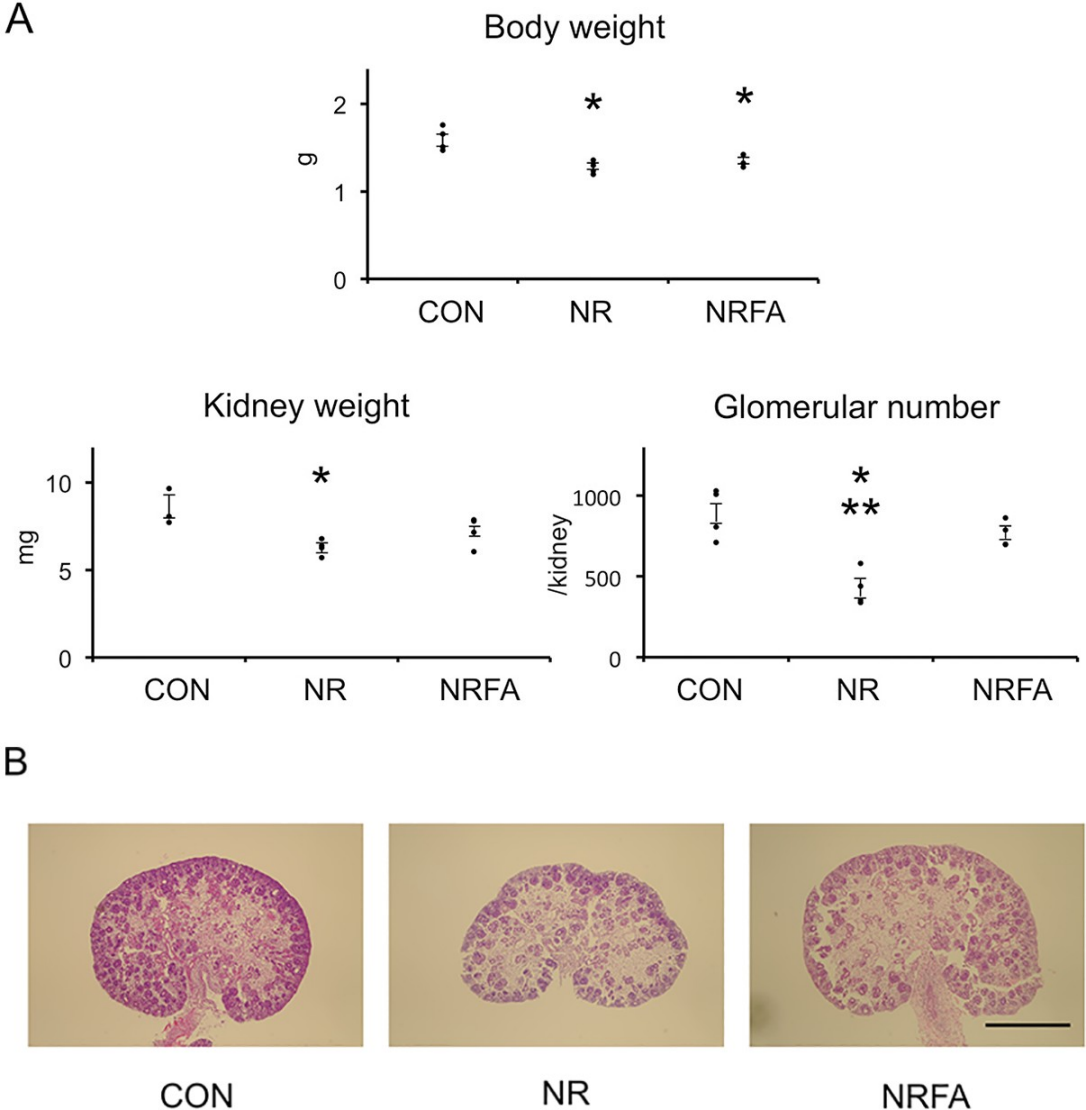
Effect of folic acid supplementation on the reduction in body weight, kidney weight, and glomerular number induced by maternal nutrient restriction at embryonic day 18. (A) After fetal weight was recorded, kidneys were isolated and weighed. They were then fixed, sectioned, and stained with hematoxylin and eosin (representative figure) or PAS (glomerular counting). Glomerular number was counted as described in Materials and Methods. The mean value (body weight and kidney weight) or one value (glomerular count) of each litter was used for statistical analysis (ANOVA followed by Tukey’s test). CON, controls; NR, offspring of nutrient-restricted mothers; NRFA, offspring of nutrient-restricted mothers supplemented with folic acid. n = 4 litters, n = 9–15 animals per litter. Dots indicate the mean value (body weight and kidney weight) or one value (glomerular number) of each litter. Bars represent ±SE. *, P<0.05 vs CON; **, P<0.05 vs NRFA. (B) Representative images. Scale bar, 1 mm.

At E18, fetal body weight, kidney weight, and glomerular number were significantly reduced by maternal nutrient restriction again in agreement with our previous report (Fig 2A). In contrast, kidney weight of fetuses from NRFA were not different from CON although body weight was significantly smaller than CON. Since it was difficult to count ureteric tip number at this stage of development, glomerular number was assessed. Glomerular number was significantly reduced in NR, but that in NRFA was not different from CON and higher than NR (Fig 2B).

### Effect of maternal nutrient restriction with or without folic acid supplementation on global DNA methylation of rat embryonic kidney

**Fig 3:**
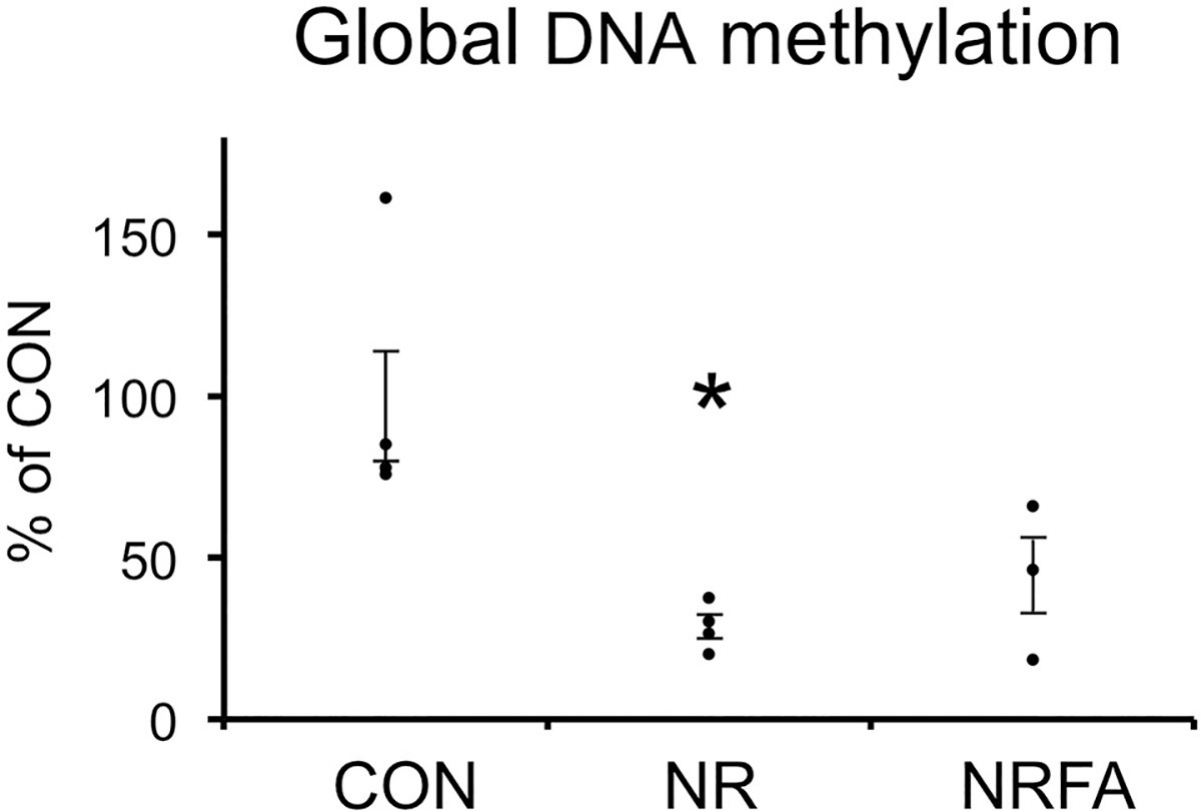
Effect of maternal nutrient restriction with or without folic acid supplementation on global DNA methylation of rat E18 embryonic kidney. CON, controls (n = 4 litters); NR, offspring of nutrient-restricted mothers (n = 4 litters); NRFA, offspring of nutrient-restricted mothers. supplemented with folic acid (n = 3 litters). n = 9–15 animals per litter. The mean value of each litter was used for statistical analysis (ANOVA followed by Tukey’s test). Dots indicate the mean value of each litter. Bars represent ±SE. *, P<0.05 vs CON.

Methylated DNA was not detected in E15 metanephroi from either CON or NR (n = 2 litters, n = 12–14 animals per litter). This was probably because the level of DNA methylation is low at this stage and because the amount of DNA obtained from the kidneys of one litter was not enough to be detected by the assay utilized. At E18, global DNA methylation was detected and significantly reduced in NR compared with CON (Fig 3). In metanephroi from NRFA, DNA methylation was not statistically different from CON, suggesting a partial restoration in DNA methylation by FA.

### Protein expression of DNA methyltransferases in the kidney from embryonic day 15

**Fig 4:**
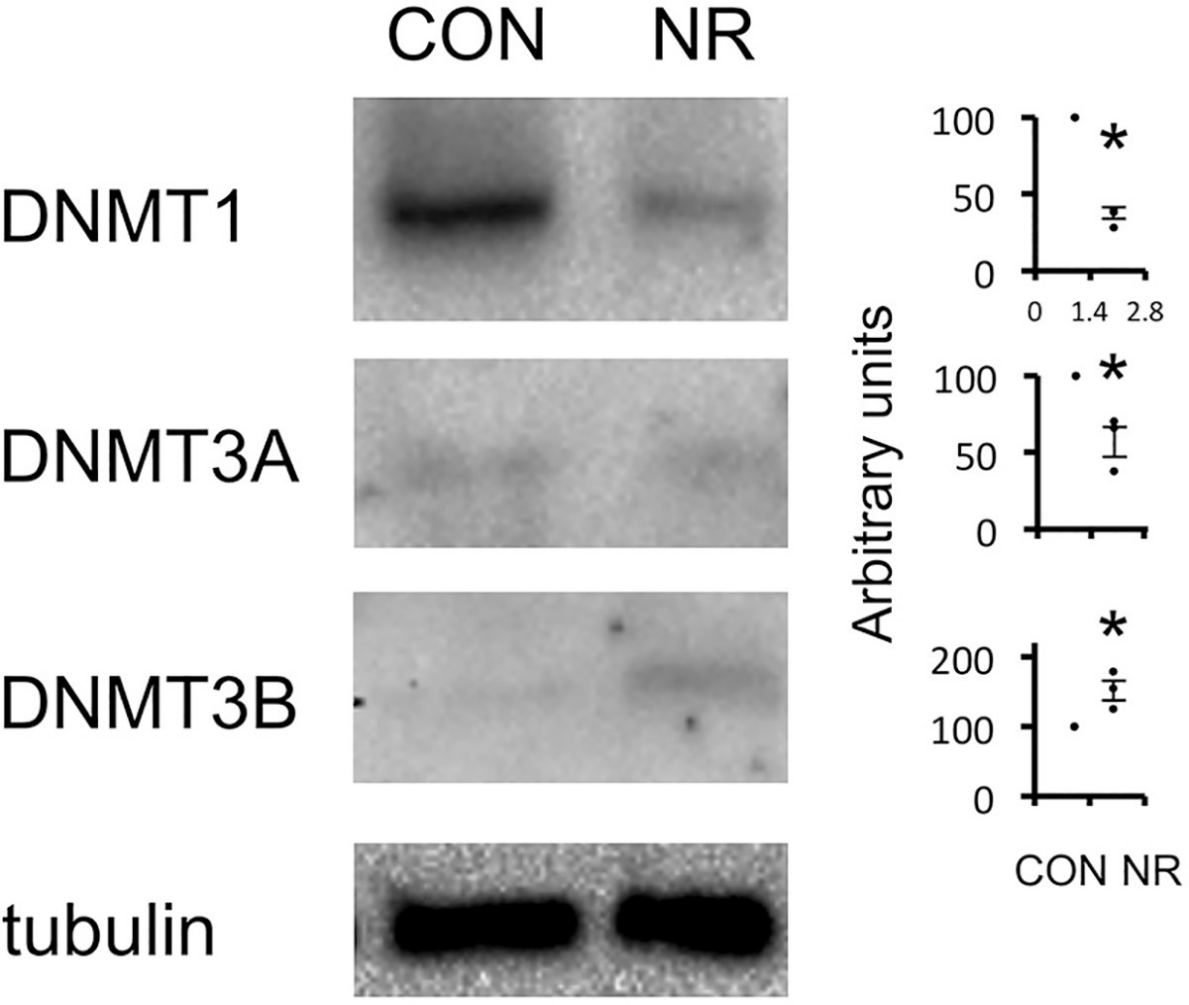
Expression of DNA methyltransferases in the kidney from embryonic day 15. (A) Representative immunoblot. (B) Quantitative analysis. n = 3 litters. n = 10–15 animals per litter. Statistical analysis was performed with unpaired *t* test. DNMT, DNA methyltransferase; CON, controls; NR, offspring of nutrient-restricted mothers. Bars represent ±SE. *, P<0.05 vs CON.

The protein expression of DNMTs was assessed in the kidney of E15 fetuses. DNMT1 was most strongly expressed, and DNMT3A and DNMT3B were weakly expressed in CON (Fig 4). In NR metanephroi, DNMT1 expression was markedly reduced. DNMT3A was also significantly reduced in NR. DNMT3B, on the other hand, was significantly upregulated in NR.

### Effect of 5-aza-2’-deoxycytidine in metanephric organ culture

**Fig 5:**
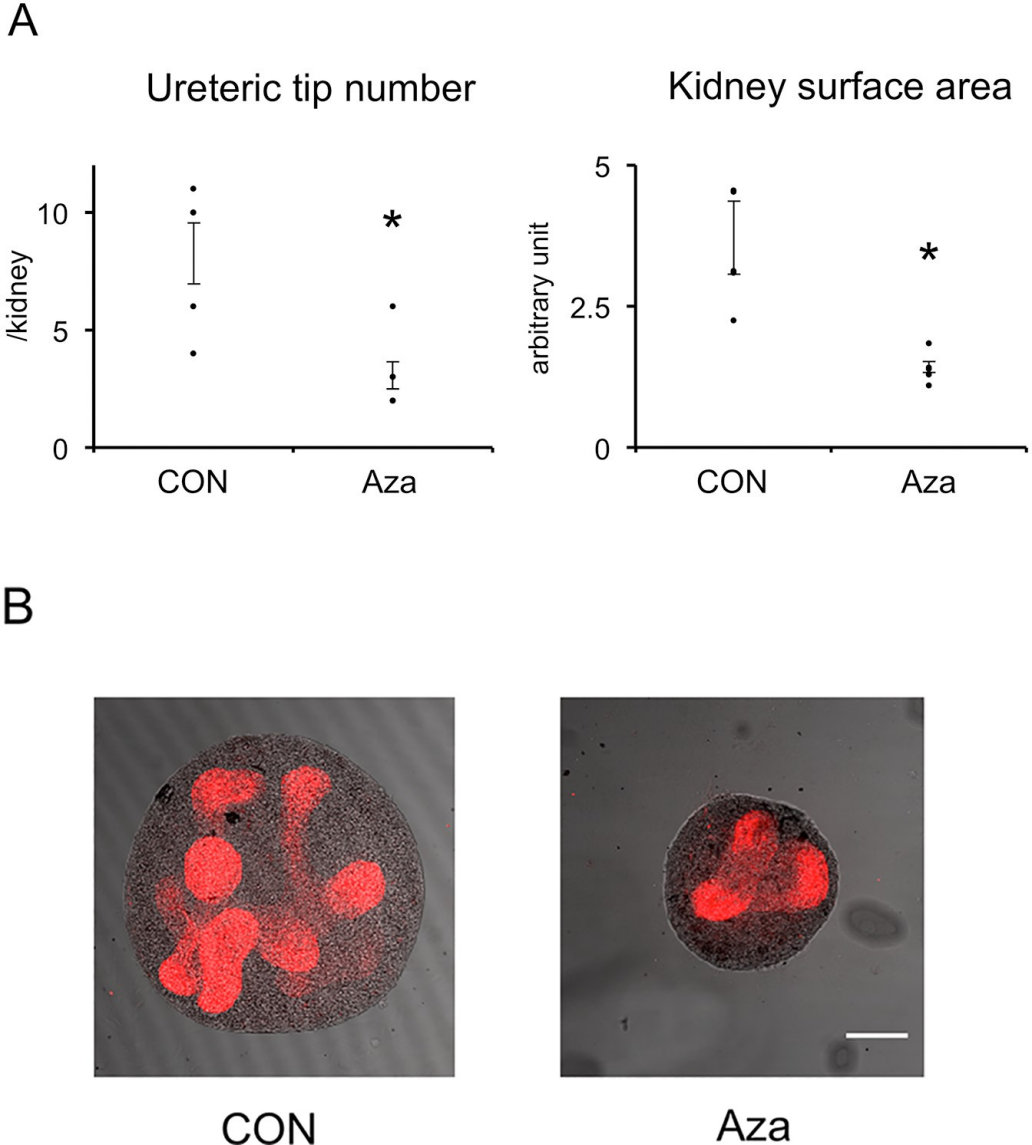
Effect of 5-aza-2’-deoxycytidine in metanephric organ culture. Embryonic day 13 metanephroi from normal dams were subjected to organ culture in the absence (CON) or presence of 5-aza-2’-deoxycytidine (Aza) for 3 days. Ureteric buds were visualized by whole mount staining with anti-pancytokeratin antibody. (A) Quantitative analysis. n = 1 litter, n = 6 kidneys. Statistical analysis was performed with ANOVA followed by Tukey’s test. Bars represent ±SE. *, P<0.05 vs CON. (B) Representative images. Scale bar, 250 **μ**m.

E13 rat metanephroi were cultured in the presence or absence of Aza, an inhibitor of DNA methylation, for 3 days. The ureteric tip number was significantly reduced in metanephroi cultured in the presence of Aza (Fig 5). The kidney surface area of Aza-treated metanephroi was also significantly reduced compared with controls. These results demonstrate the importance of DNA methylation in ureteric branching and metanephric growth.

### Effect of folic acid deficient- and methyl donor-deficient medium in metanephric organ culture

**Fig 6:**
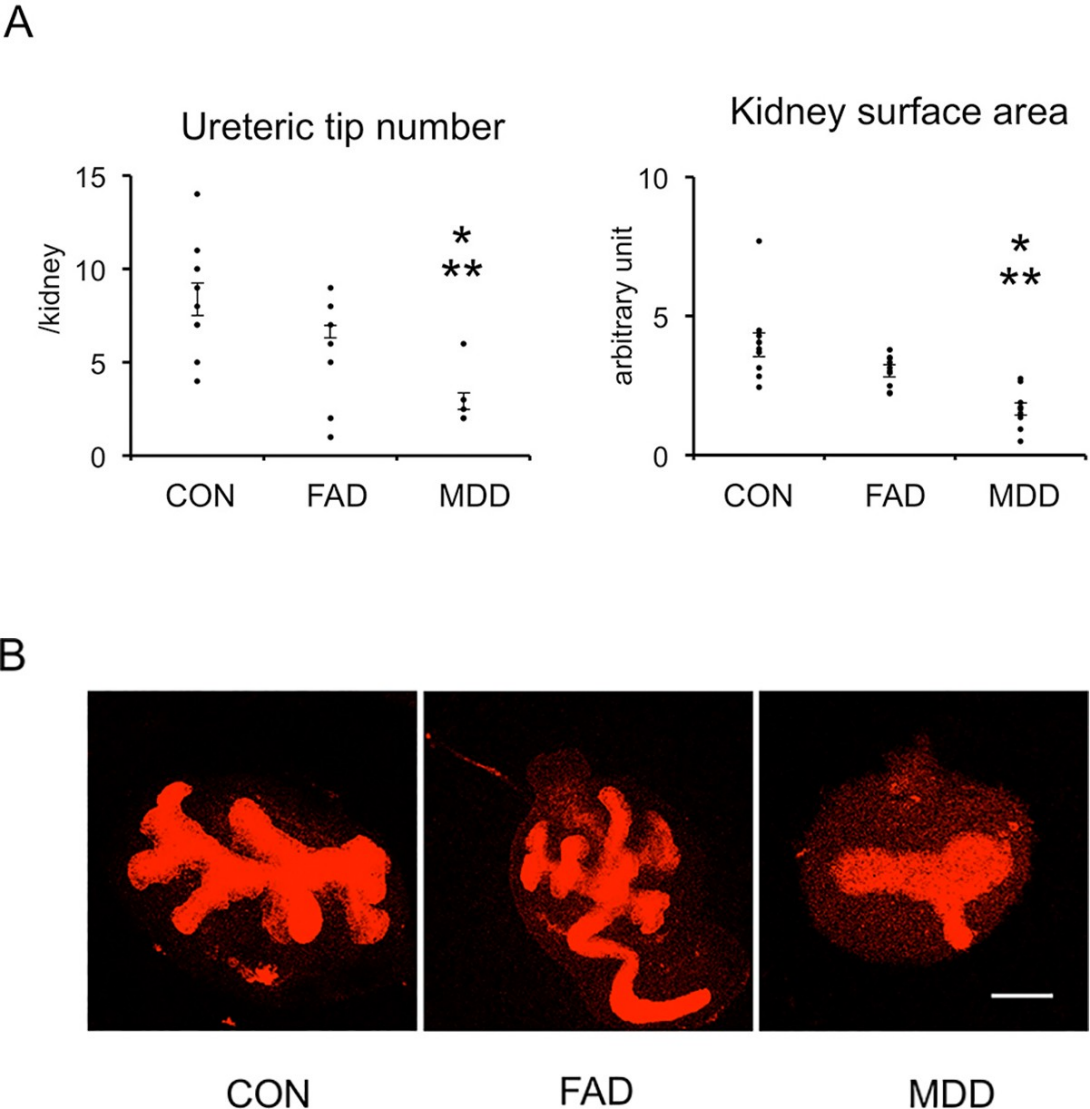
Effect of folic acid deficient- and methyl donor-deficient medium in organ culture. Embryonic day 13 metanephroi from normal rats were subjected to organ culture in DMEM (CON), DMEM deficient in FA (FAD), or DMEM deficient in FA, choline, and methionine (MDD) for 3 days. Ureteric buds were visualized by whole mount staining with anti-pancytokeratin antibody. **(**A) Quantitative analysis. n = 3 litters, n = 10–11 kidneys. Statistical analysis was performed with ANOVA followed by Tukey’s test. Bars represent ±SE. *, P<0.05 vs CON; **, P<0.05 vs FAD. **(**B) Representative images. Scale bar, 250 μm.

E13 rat metanephroi were cultured in DMEM, FA-deficient medium (FAD), or DMEM lacking FA, choline, and methionine (methyl donor-deficient medium, MDD) (Fig 6). Metanephroi cultured in FAD tended to have a lower number of ureteric tips and be smaller in size than those cultured in control medium although statistical significance was not achieved. Ureteric bud tip number and kidney surface area of metanephroi cultured in MDD were significantly reduced compared with controls and those cultured in FAD. The effect of MDD was similar to that of Aza mimicking NR.

## Discussion

In the present study, we found that FA supplementation attenuated the inhibitory effect of maternal nutrient restriction on ureteric branching, kidney growth, and glomerular formation. The effect of FA supplementation was associated with restoration of reduced DNA methylation in the E18 kidney. *In vitro* studies demonstrated that DNA methylation is required for metanephric development. Furthermore, *in vitro* methyl donor deficiency reproduced the effects of NR. These results suggest that the effect of FA supplementation involves DNA methylation.

Supplementation of FA to nutrient restricted rat dams has been reported to prevent hypertension in the offspring [8]. The mechanism, however, has not been elucidated. We hypothesized that the prevention of hypertension by FA could be through an effect on nephron number, which is known to be reduced by maternal low protein diet [1]. In our model of maternal nutrient restriction as well, nephron number was reduced along with the inhibited ureteric branching [6]. Since nutrients were reduced to 50% in our model, it is likely that the adverse effect on kidney development is through, at least in part, the deficiency of methyl donors including FA. Dietary supplementation with FA alleviated the reduced ureteric bud branching induced by maternal nutrient restriction at an earlier stage (E14). At this stage, kidney size was not reduced in NR or in NRFA. At later stage of development (E18), reduced glomerular number in NR was alleviated by FA supplementation as well as reduced kidney weight.

FA has been recognized to be necessary for normal development and its maternal supplementation lowers the risk of various congenital anomalies of central nervous system, heart, kidney, and others. Maternal FA supplementation has also been reported to alleviate the effect of adverse intrauterine environment including maternal nutrient or protein restriction, high fat diet, and alcohol, on various organs including liver, muscle, central nervous system, and heart [14, 15]. A systematic review in animals and humans reported that obesity and insulin resistance in the offspring may also be influenced by maternal FA status [16]. The mechanism of FA is thought to be linked to DNA methylation.

DNA methylation is an important mechanism modulating gene expression, and many previous studies reported an association with adverse intrauterine conditions [17, 18]. In rats, maternal uterine artery ligation decreased DNA methylation in the liver of postnatal offspring [19]. Maternal nicotinamide caused DNA hypomethylation of fetal liver [20]. The change in global DNA methylation was shown to occur in the offspring of baboons subjected to nutrient restriction during pregnancy in an organ- and developmental stage-specific manner [7]. Thus in the fetal kidney, global methylation was reduced and increased at 0.5 and 0.9 gestation, respectively, whereas methylation was unchanged in the liver at either time point. The effect of a global change in DNA methylation was proposed to be two-fold [7]. One is reduction in methylation of specific genes that results in the change in gene activity. The second is effects on genomic organization and overall genome function. Decreased global methylation has been thought to contribute to genomic instability [21] and is associated with predisposition to pathological states and the development of diseases including cancer, lupus, and congenital anomaly [22, 23]. In support of this notion, DNMT1 knock out mice presented with genome wide organizational defects and embryonic lethality [24].

Supplementation with methyl donors including FA has been reported to alleviate the effects of adverse intrauterine environment [25]. In only a few studies, however, DNA methylation status has been examined. Carlin et al reported that the supplementation with methyl donors including FA inhibited the adverse effects of maternal high fat diet in the central nervous system, which was associated with attenuation of DNA hypomethylation [26]. In normal rats, maternal FA supplementation has been shown to modulate global DNA methylation of the offspring in an organ-specific manner [27]. In the kidney, DNA methylation was not altered contrasting to the current study, probably because the effect of FA is different in normal and adverse maternal environment.

DNMTs are enzymes that have a central role in DNA methylation. It is generally agreed that DNMT3 establishes DNA methylation and that DNMT1 maintains it [28]. Loss of DNMT1 results in global DNA hypomethylation and embryonic lethality, whereas deletion of DNMT3A and DNMT3B results in impaired postnatal development and embryonic lethality, respectively [24, 29]. DNMTs were expressed in both fetal and adult tissues in an organ-specific manner [30]. In postnatal piglets with spontaneous intrauterine growth restriction, the expression of DNMT1 in the ileum was reported to be reduced [31]. Maternal FA supplementation to the same model reversed the decreased DNMT1 expression in the liver [32]. The effect of FA in the present study may be through upregulation of DNMT1. We leave this hypothesis for future work. DNMT1 was also reduced in the postnatal liver of IUGR rats induced by maternal uterine artery ligation, which was associated with reduced DNA methylation [19]. In the kidney, decreased mRNA expression of DNMT1 has been reported in neonatal IUGR rats induced by maternal uterine artery ligation [3]. This was associated with decreased p53 promoter methylation and increased p53 mRNA. In the liver of offspring of protein-restricted rats, the expression of DNMT1 was decreased, whereas DNMT3A and DNMT3B were unaltered [33]. These changes were associated with hypomethylation of hepatic glucocorticoid receptor. In the same study, reduced DNMT1 expression was restored by maternal FA supplementation but the expression of DNMT3A and DNMT3B was again unchanged [33]. ChIP assays using an anti-DNMT1 antibody showed that binding of DNMT1 at the promoter of glucocorticoid receptor was significantly lower in the protein-restricted offspring compared with controls. The authors proposed the importance of DNMT1 rather than DNMT3s in maternal protein restriction offspring. The strong expression of DNMT1 in CON as well as its reduction in NR in the E15 kidney shown in the present study is compatible with their view.

While this manuscript was in preparation, two important papers have been published. Both reported that DNMT1 in nephron progenitor cell is important for kidney development [12, 34]. Deletion of *Dnmt1*, but not *Dnmt3a or Dnmt3b*, resulted in reduction of global DNA methylation, nephron number, and kidney size. Two models of IUGR, *in vitro* culture of metanephroi under high-glucose conditions and maternal uterine artery ligation, showed decreased global DNA methylation, nephron number, and kidney size [12]. DNMT1 was expressed predominantly in the cap mesenchyme and nephron precursors [34]. Expression of all DNMTs decreased after birth [34]. These results are in accordance with our findings.

We examined the role of DNA methylation in kidney development using organ culture. Aza, an inhibitor of DNA methylation, inhibited ureteric branching and kidney growth, in resemblance to the effect of maternal nutrient restriction. We further investigated the effect of methyl donor deficient-medium. While FA is an important co-factor in one-carbon metabolism, methionine and choline are the major sources of methyl groups. Medium lacking these nutrients inhibited ureteric bud branching and kidney growth in a similar manner to Aza. The more severe effect of Aza and MDD compared with maternal nutrient restriction could be that Aza- and MDD decreased DNA methylation more. Maternal nutrient restriction reduces but does not completely deplete methyl donors. Taken together, these *in vitro* results reproduced the effect of maternal nutrient restriction, underscoring the importance of global DNA methylation in kidney development.

## Conclusion

FA supplementation to nutrient restricted rat dams alleviates the reduced ureteric branching, kidney growth, nephrogenesis, and DNA methylation in the offspring. DNA methylation is necessary for metanephric development and is likely to be involved in the impaired kidney development by NR. These findings may provide a basis for preventive measures for chronic kidney disease programmed by an adverse intrauterine environment.

## Supporting information

S1 Fig (TIF)

S2 Fig (TIF)

S3 Fig (JPG)

S4 Fig (TIF)

S5 Fig (TIF)

S6 Fig (TIF)

S7 Fig (TIF)

S8 Fig (TIF)

S9 Fig (TIF)

S10 Fig (TIF)

S11 Fig (TIF)

S12 Fig (TIF)

S13 Fig (TIF)

S14 Fig (JPG)

S15 Fig (JPG)

S1 Dataset (XLSX)
