# Supplementary figures and images for: Folic acid supplementation alleviates reduced ureteric branching, nephrogenesis, and global DNA methylation induced by maternal nutrient restriction in rat embryonic kidney
Source: PLoS One. 2020 Apr 6;15(4):e0230289. doi: 10.1371/journal.pone.0230289 (PMC7135271; doi:10.1371/journal.pone.0230289)

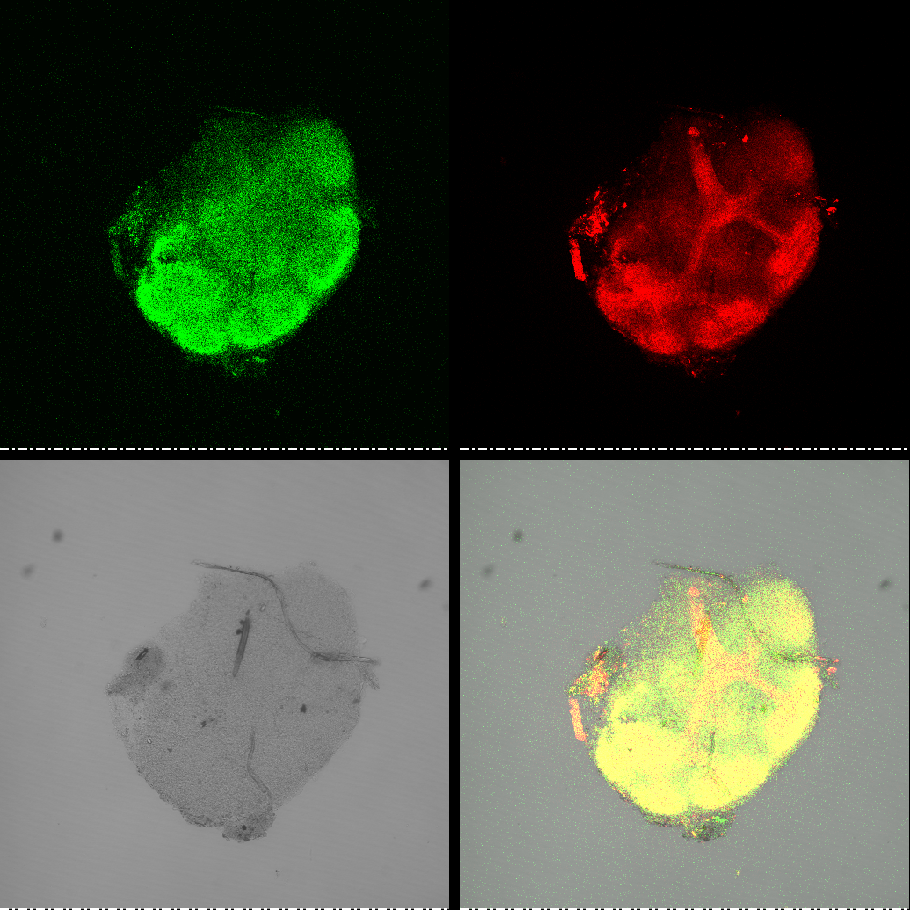

Supplement: S1 Fig — (TIF) [file pone.0230289.s001.tif]

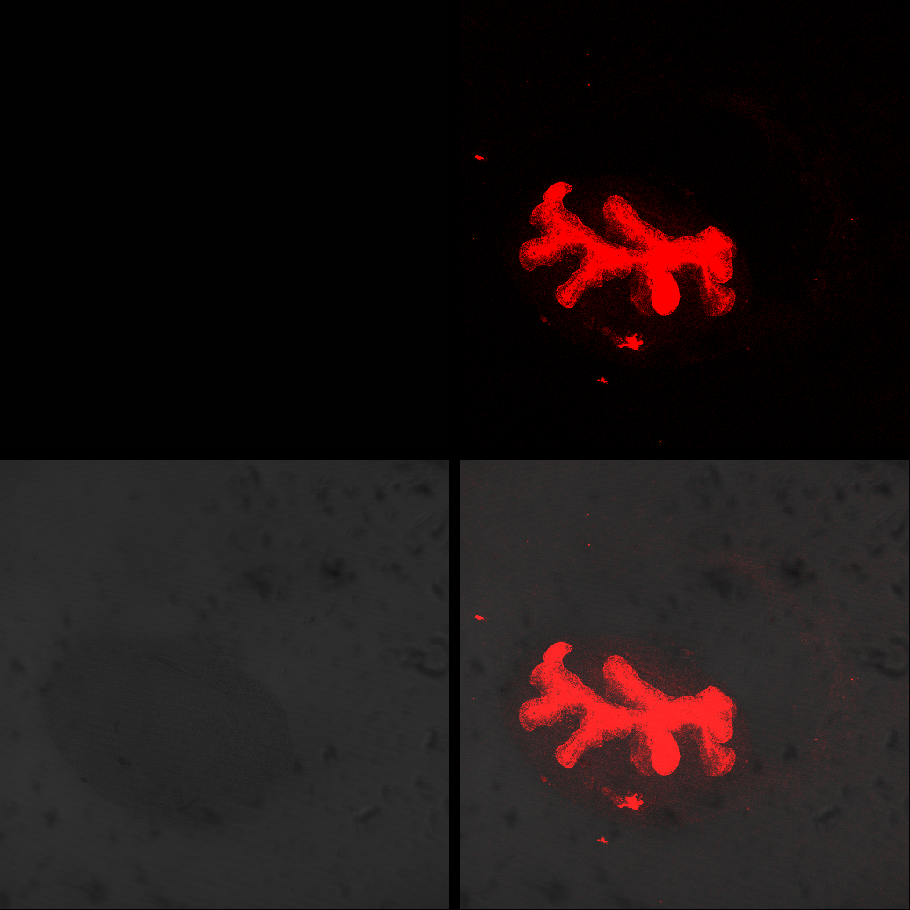

Supplement: S2 Fig — (TIF) [file pone.0230289.s002.tif]

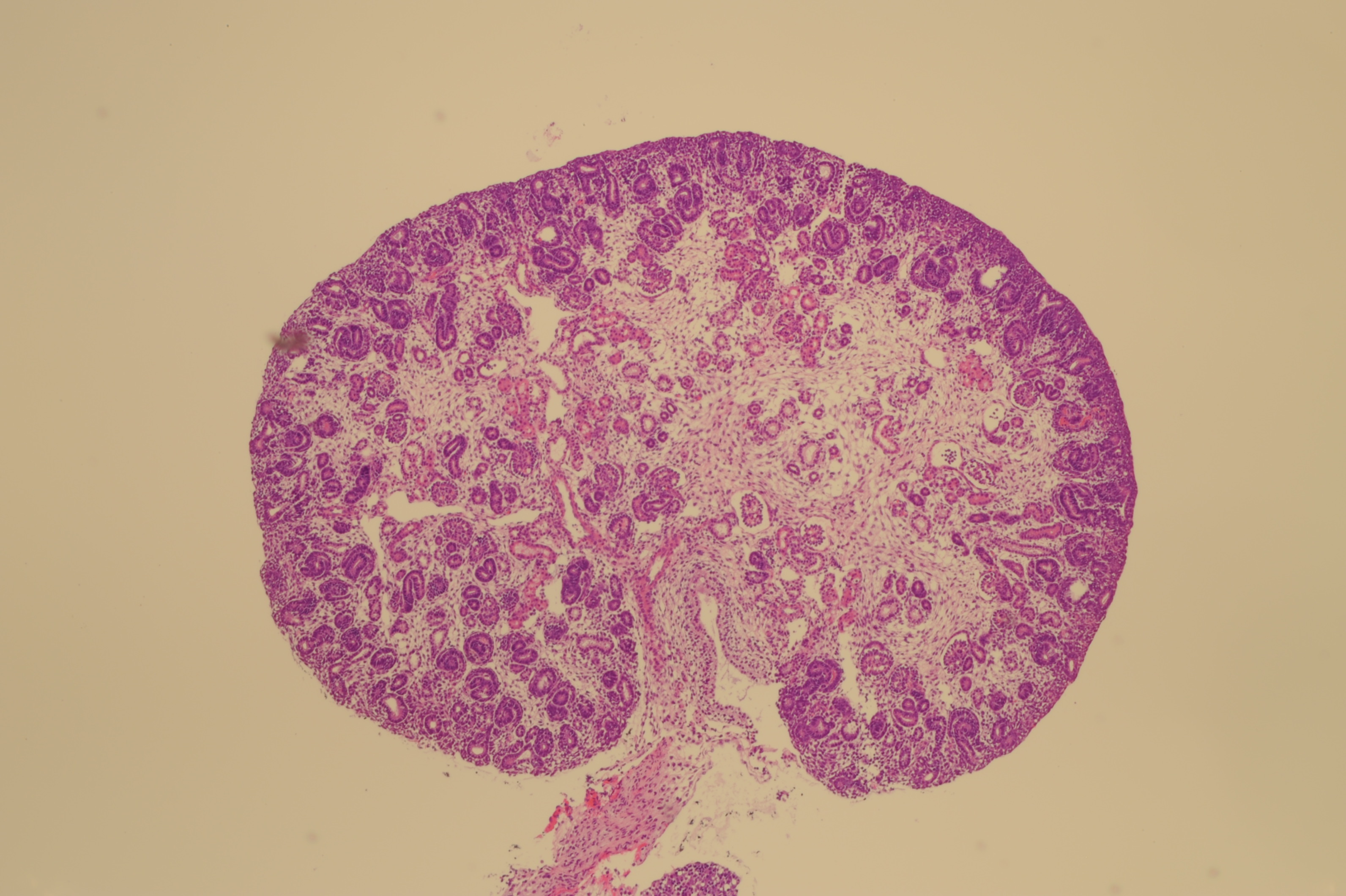

Supplement: S3 Fig — (JPG) [file pone.0230289.s003.jpg]

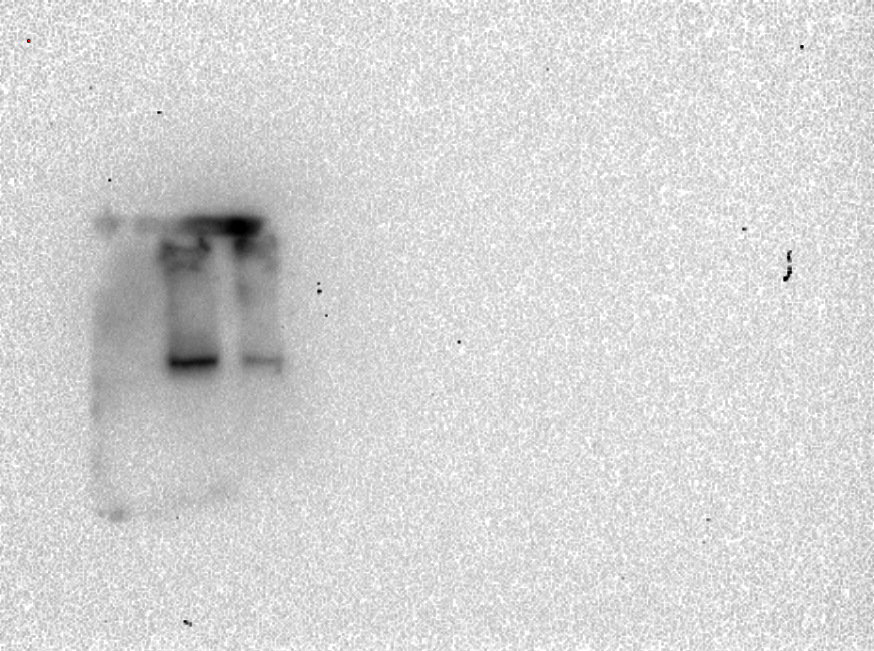

Supplement: S4 Fig — (TIF) [file pone.0230289.s004.tif]

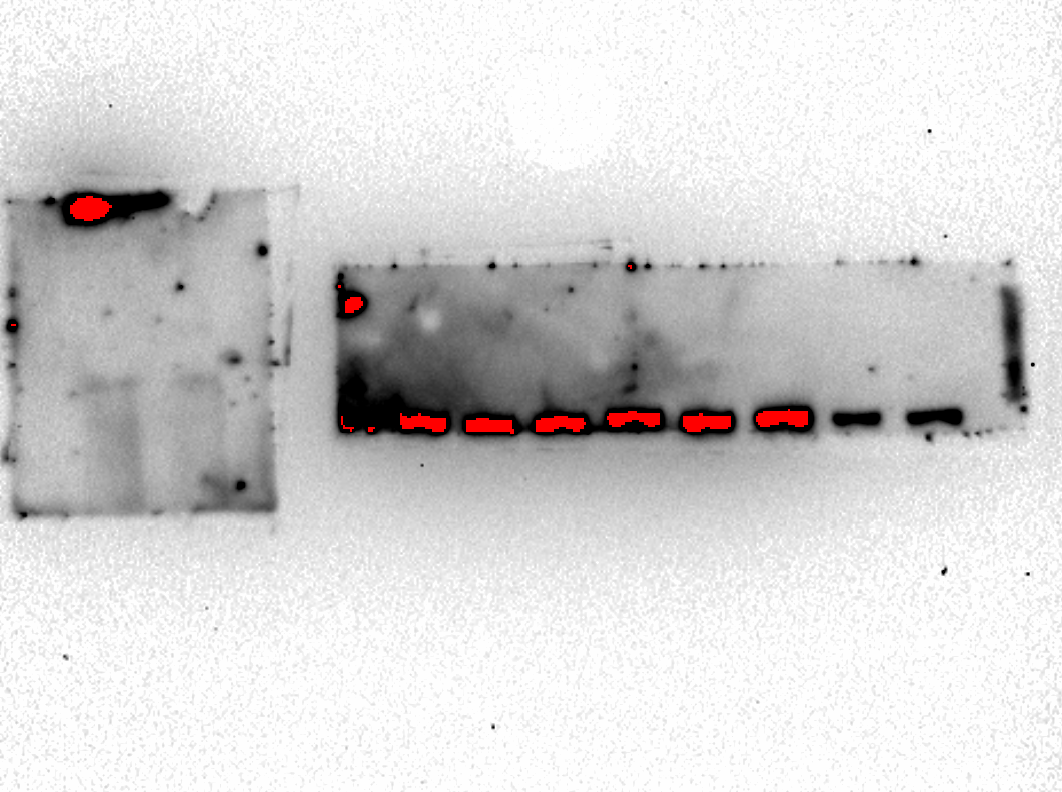

Supplement: S5 Fig — (TIF) [file pone.0230289.s005.tif]

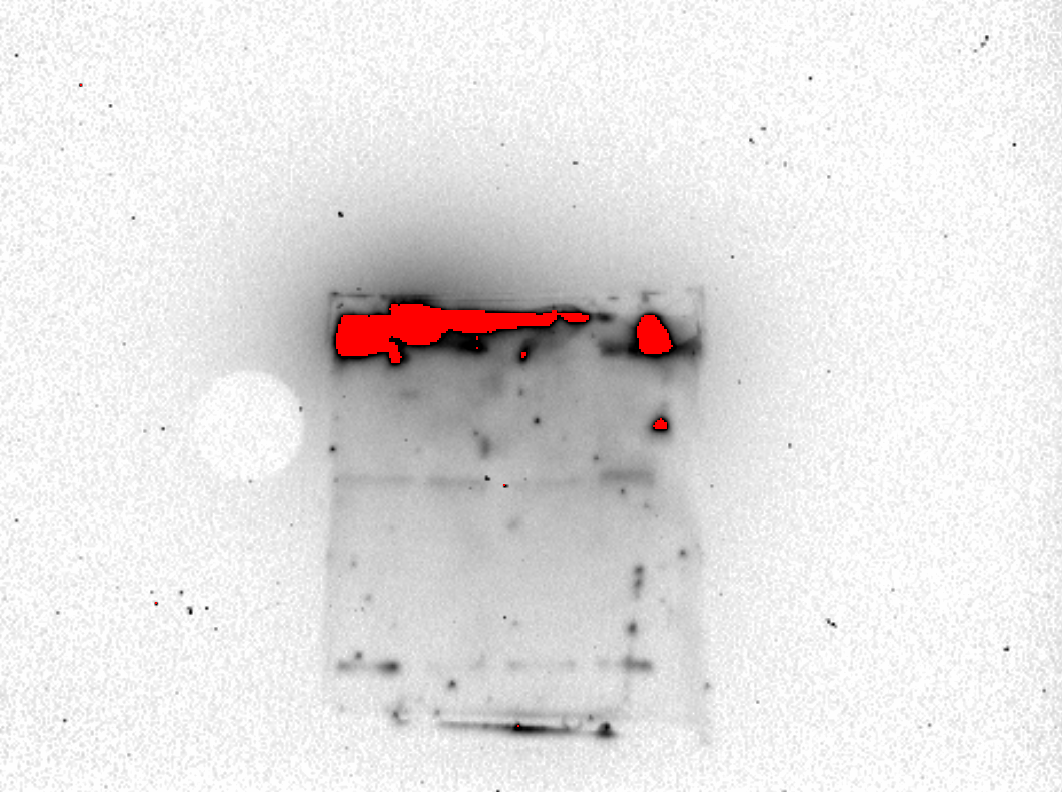

Supplement: S6 Fig — (TIF) [file pone.0230289.s006.tif]

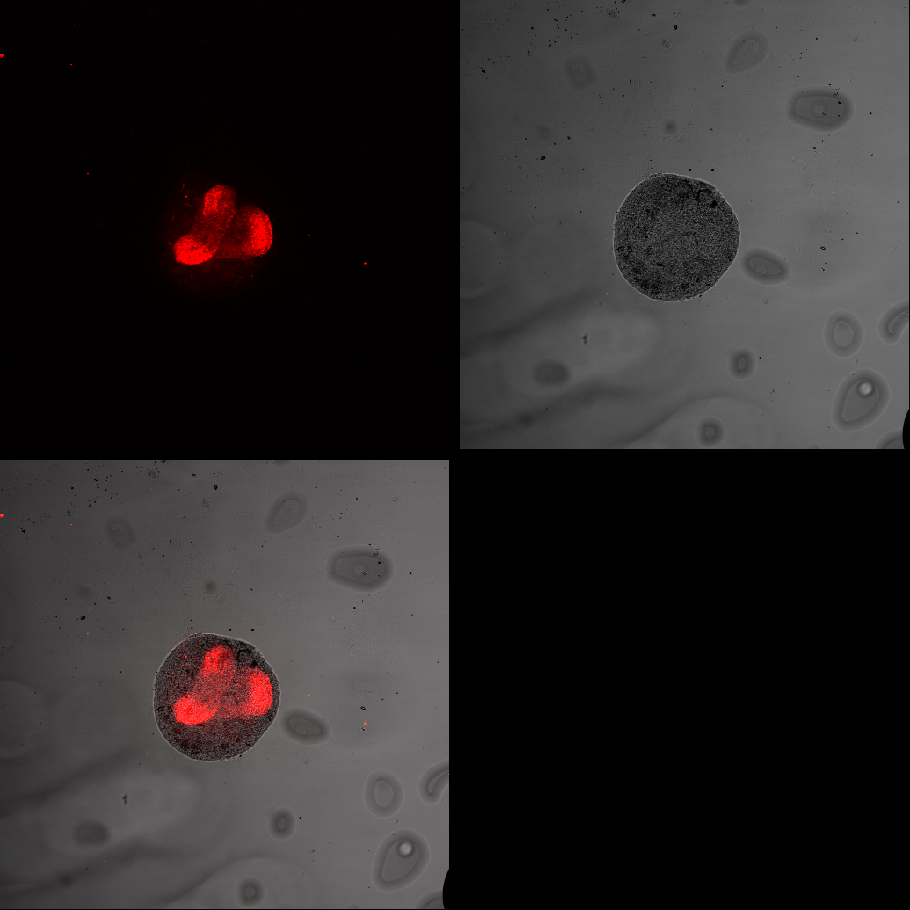

Supplement: S7 Fig — (TIF) [file pone.0230289.s007.tif]

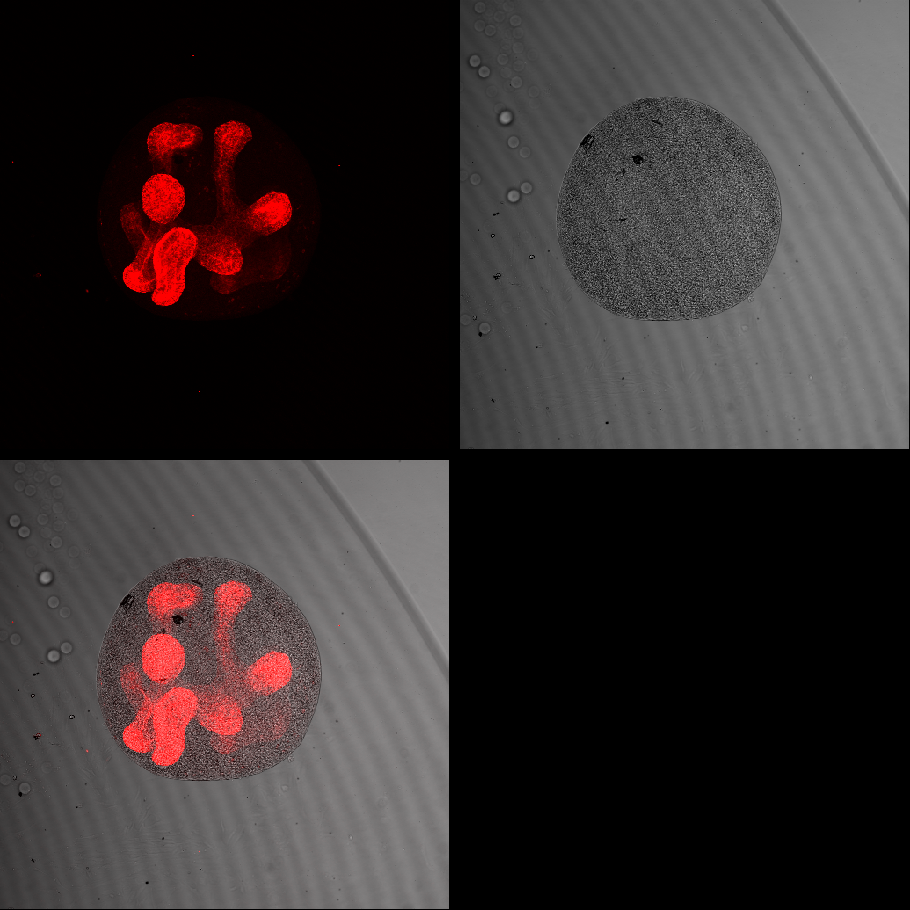

Supplement: S8 Fig — (TIF) [file pone.0230289.s008.tif]

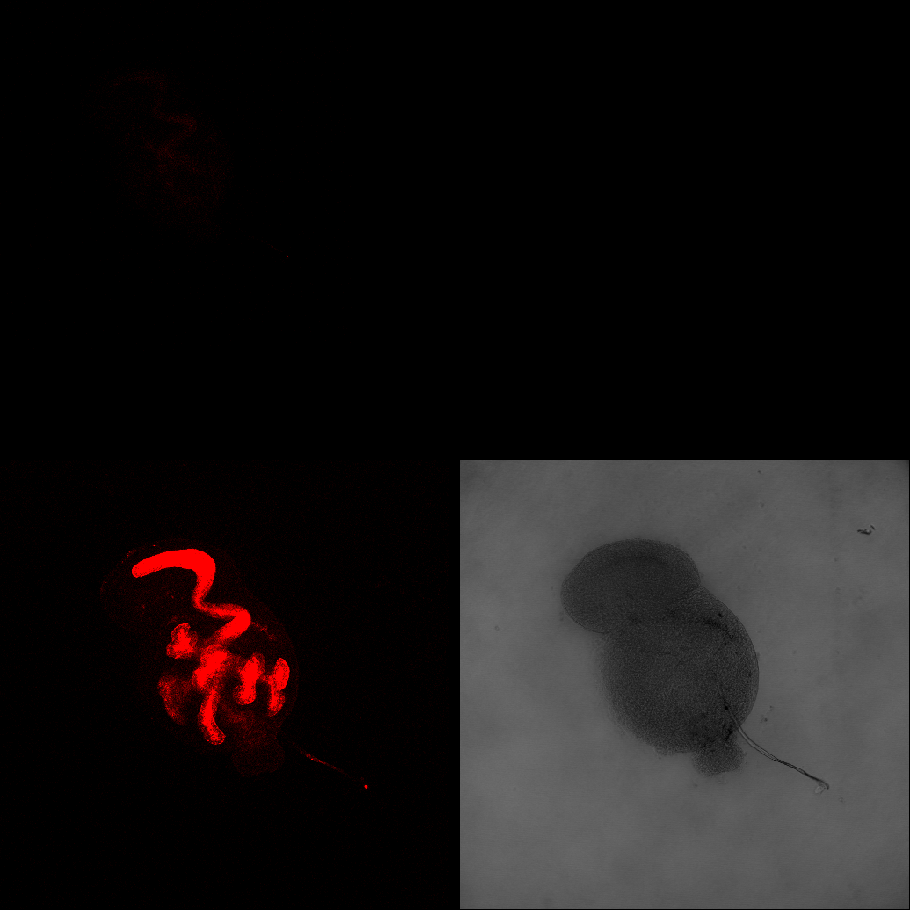

Supplement: S9 Fig — (TIF) [file pone.0230289.s009.tif]

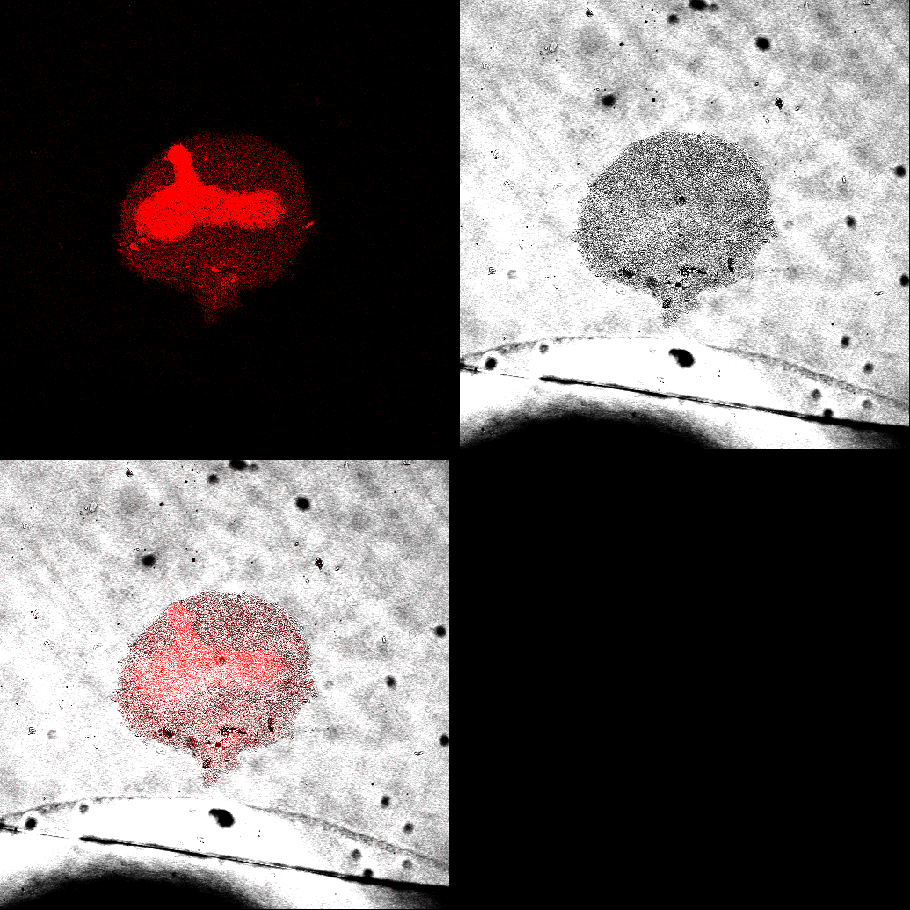

Supplement: S10 Fig — (TIF) [file pone.0230289.s010.tif]

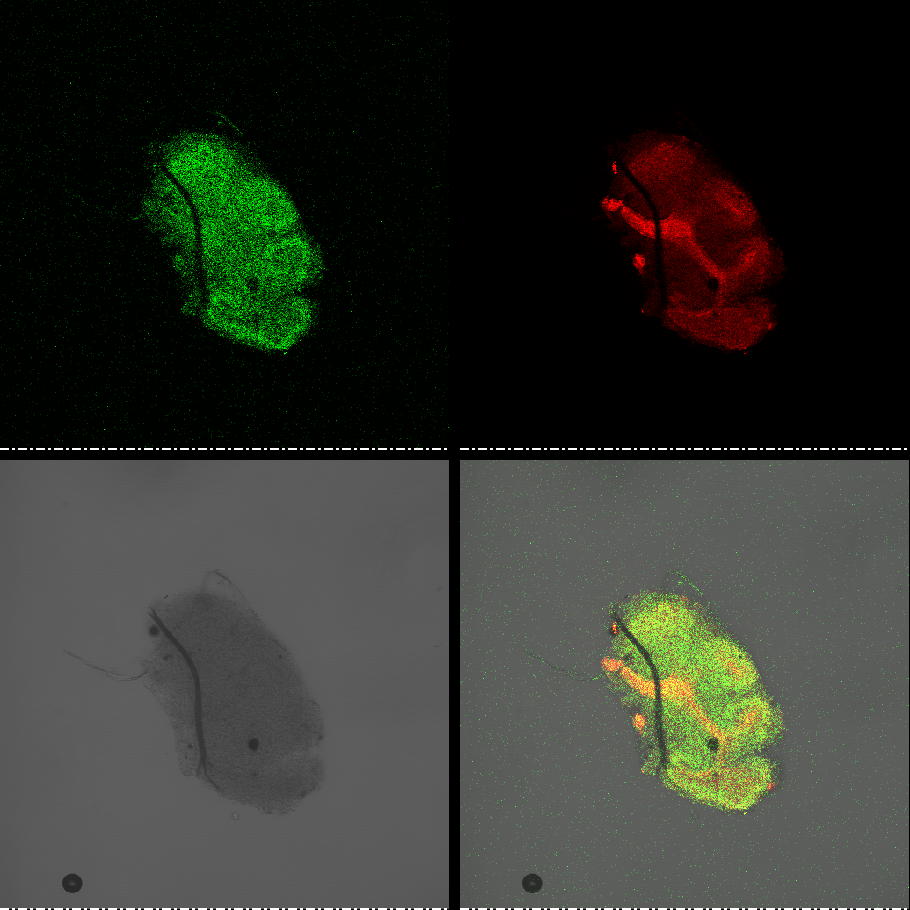

Supplement: S11 Fig — (TIF) [file pone.0230289.s011.tif]

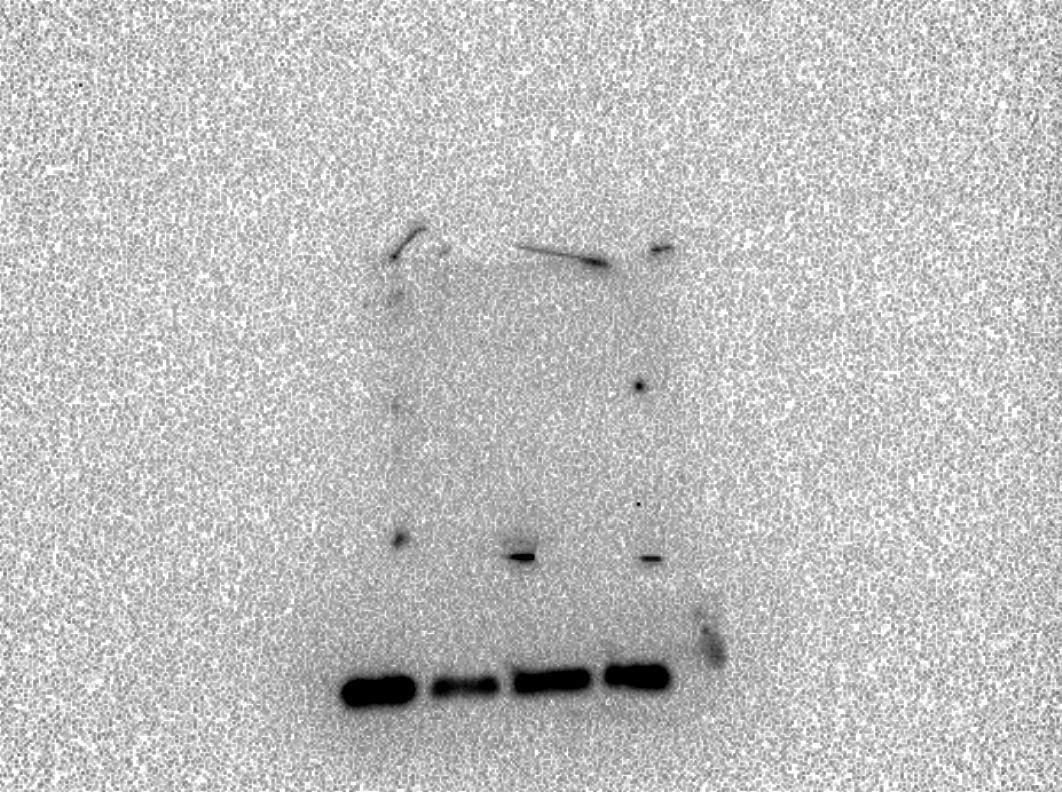

Supplement: S12 Fig — (TIF) [file pone.0230289.s012.tif]

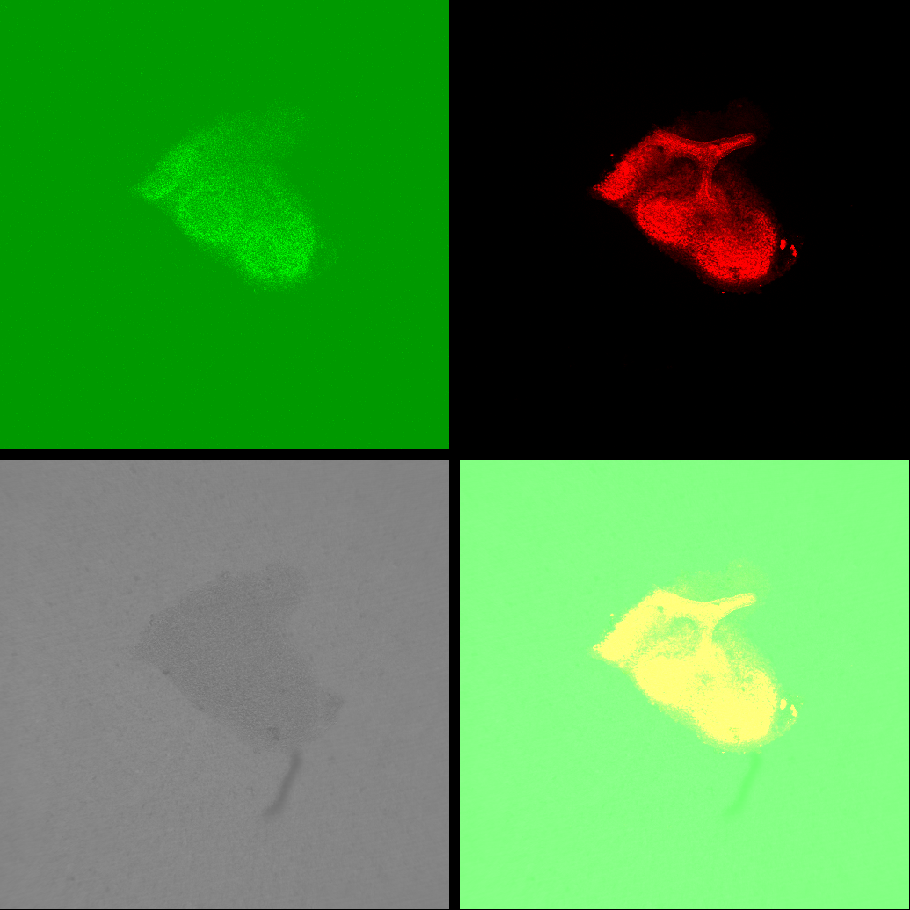

Supplement: S13 Fig — (TIF) [file pone.0230289.s013.tif]

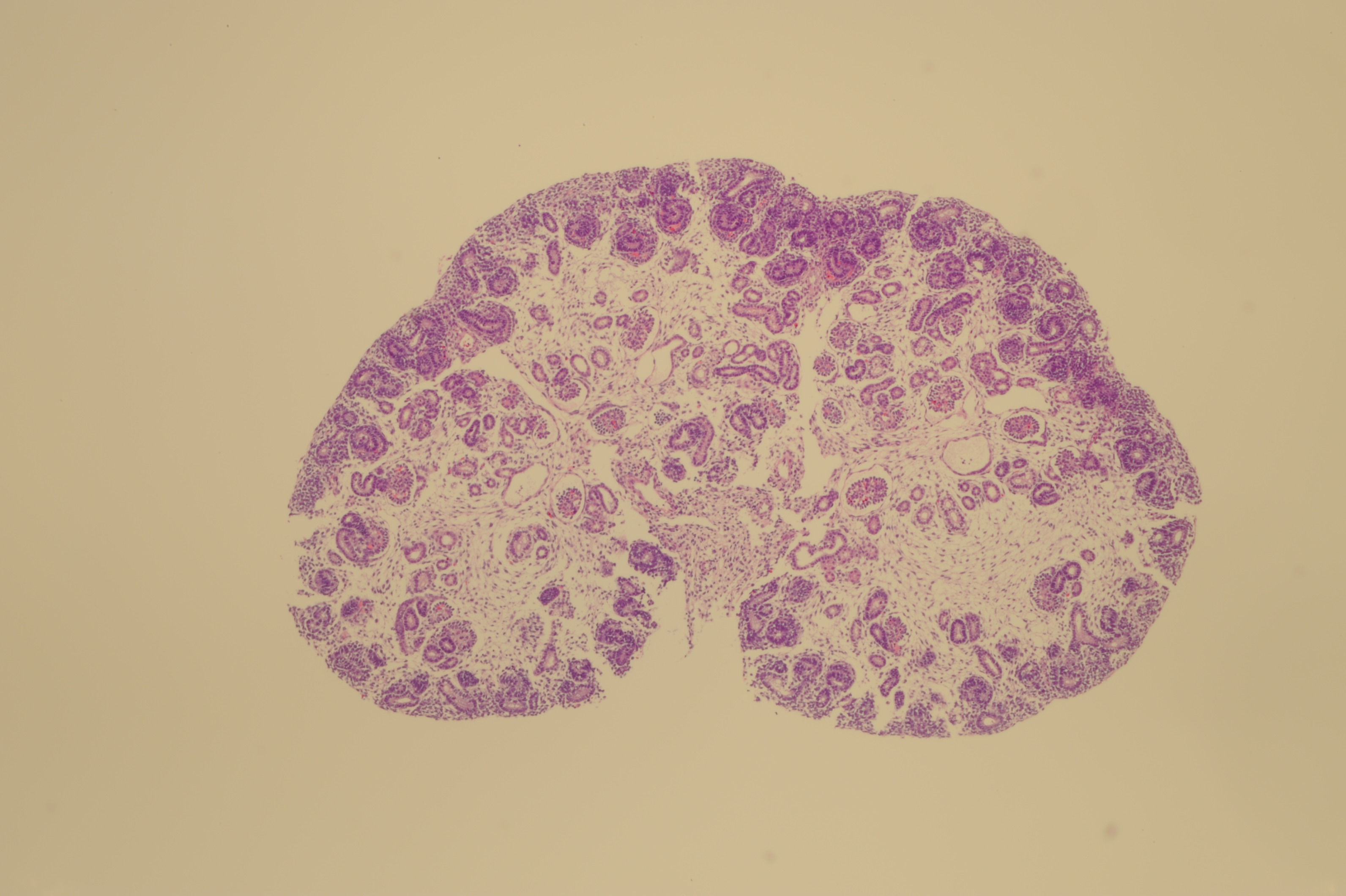

Supplement: S14 Fig — (JPG) [file pone.0230289.s014.jpg]

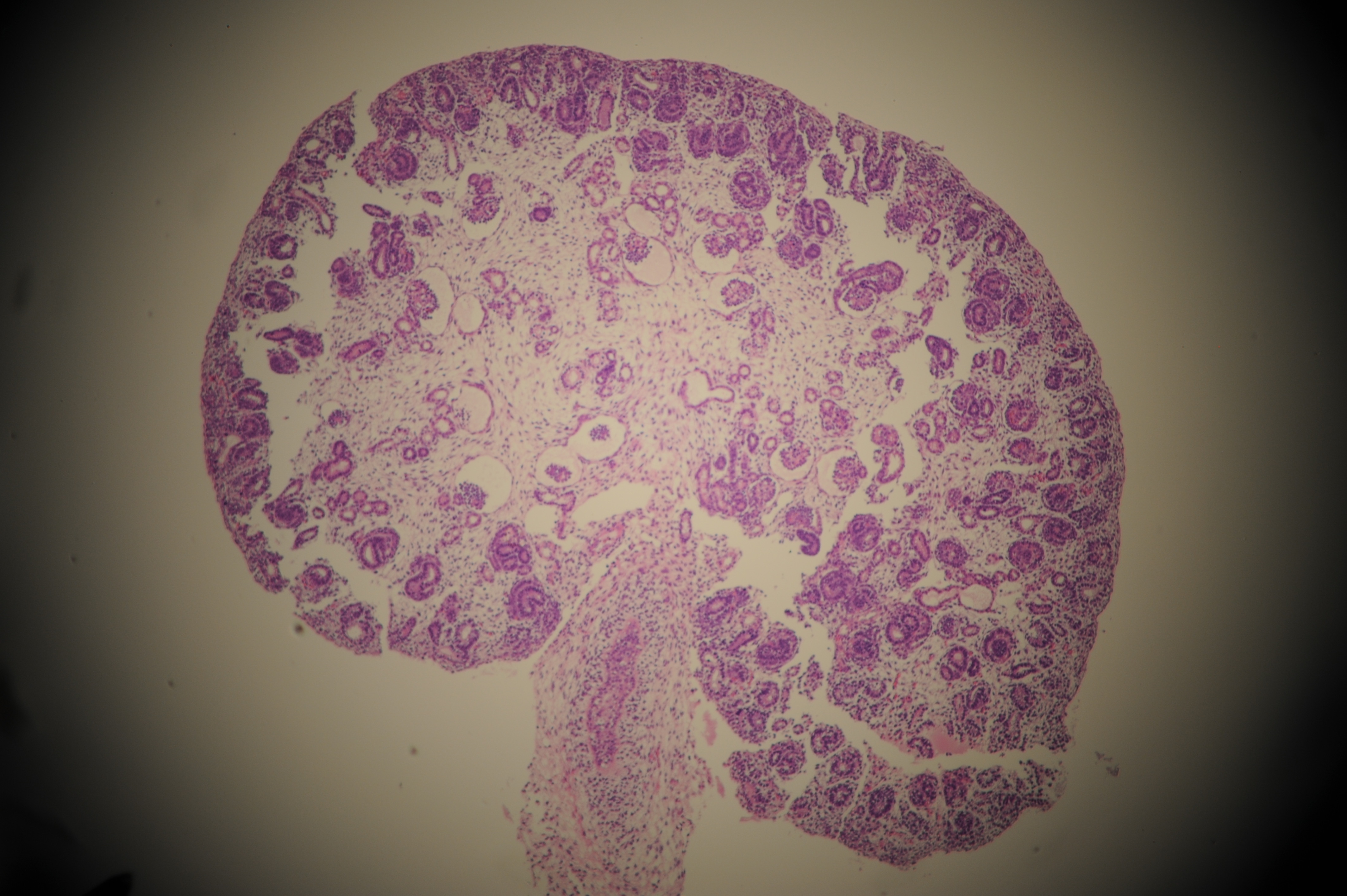

Supplement: S15 Fig — (JPG) [file pone.0230289.s015.jpg]
